# Unfolding the Role of Stress Response Signaling in Endocrine Resistant Breast Cancers

**DOI:** 10.3389/fonc.2015.00140

**Published:** 2015-06-22

**Authors:** Robert Clarke, Katherine L. Cook

**Affiliations:** ^1^Department of Oncology, Lombardi Comprehensive Cancer Center, Georgetown University Medical Center, Washington, DC, USA

**Keywords:** unfolded protein response, glucose regulated protein 78, X-box binding protein 1, estrogen receptor-α, tamoxifen, ICI 182,780, antiestrogen resistant breast cancer

## Abstract

The unfolded protein response (UPR) is an ancient stress response that enables a cell to manage the energetic stress that accompanies protein folding. There has been a significant recent increase in our understanding of the UPR, how it integrates physiological processes within cells, and how this integration can affect cancer cells and cell fate decisions. Recent publications have highlighted the role of UPR signaling components on mediating various cell survival pathways, cellular metabolism and bioenergenics, and autophagy. We address the role of UPR on mediating endocrine therapy resistance and estrogen receptor-positive breast cancer cell survival.

## The Unfolded Protein Response and Endoplasmic Reticulum Stress

**Figure 1:**
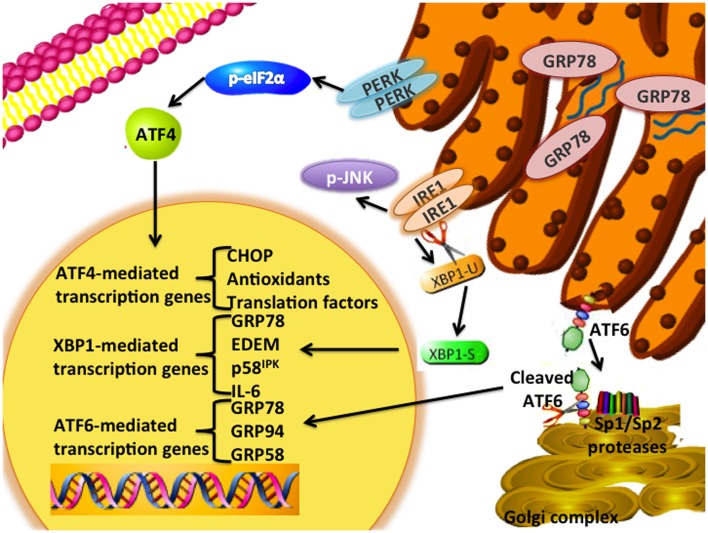
**UPR signaling schematic**. The UPR signaling pathway is an adaptive endoplasmic reticulum response. GRP78 unbinds from the three signaling arms (IRE1, PERK, ATF6) upon sensing the accumulation of unfolded proteins within the lumen of the endoplasmic reticulum. PERK is a type I transmembrane protein that phosphorylates the eukaryotic translation initiation factor 2 α-subunit (eIF2α). Induction of ATF4 regulates the expression of several genes, including the pro-apoptotic DNA damage-inducible transcript 3 (DDIT3; referred to as CHOP) and antioxidant signaling responses. ATF6 is a type-II transmembrane bZIP transcription factor in which Golgi localization signals are blocked by GRP78 binding. Dissociation of GRP78 from ATF6 results in translocation of ATF6 to the Golgi complex, and cleavage of ATF6 by the site-1 (SP1) and -2 proteases (SP2) to form its active p50 form. Several downstream targets of ATF6 include GRP78, GRP94, and GRP58. Activation of IRE1α enables the unconventional splicing of XBP1. Various downstream targets of XBP1(S) include p58^IPK^, ERAD signaling components, and several protein chaperones.

**Figure 2:**
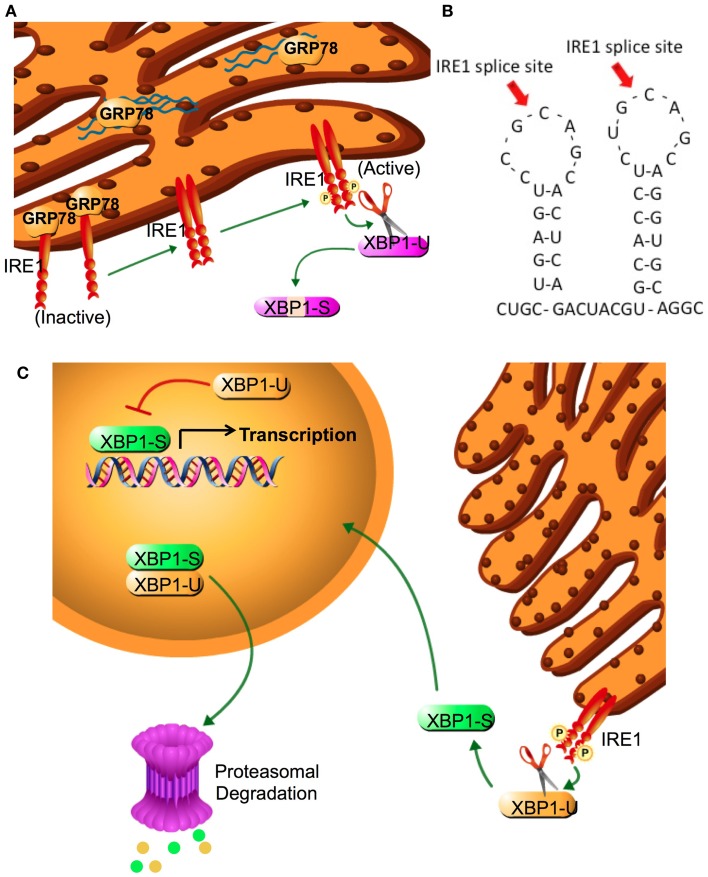
**IRE1 activation and XBP1 splicing**. **(A)** XBP1 splicing is performed by IRE1α, a type II transmembrane EnR protein with both serine-threonine kinase and endonuclease activities. The endoplasmic reticulum luminal domain of IRE1α regulates its kinase function, which is required to activate the endonuclease. In the absence of an activated UPR, IRE1-α is inactive and bound to GRP78. Following sensing of the UPR, IRE1α dissociates from GRP78 and two luminal domains form a symmetric dimer that facilitates *trans-*autophosphorylation This phosphorylation promotes nucleotide binding, activating the endonuclease domain. **(B)** Amino acid two stem-loop structure of XBP1. IRE1 cleavage sites at the two CNGNNG motifs of XBP1 are indicated by arrows. **(C)** IRE1α removes a 26-base intron sequence from XBP1 producing a translational frameshift. Splicing of the XBP1 mRNA creates the transcriptional activation domain and eliminates the nuclear exclusion and degradation domains encoded in XBP1(U). Both XBP1(S) and XBP1(U) are translated into protein; each retains the DNA binding domain and can form XBP1(S)/XBP1(U) heterodimers. The nuclear exclusion domain in the XBP1(U) partner may keep the heterodimer extranuclear, whereas its degradation sequence may target both partners for degradation. Thus, XBP1(U) acts as an endogenous dominant negative of XBP1(S). Cytoplasmic splicing of existing XBP1(U) allows for a rapid and efficient response to cellular stress that does not require additional XBP1 transcription. Since unspliced mRNA is eventually degraded, adaptation to prolonged stress requires new transcription of XBP1(U) and its splicing to XBP1(S).

The unfolded protein response (UPR) is an endoplasmic reticulum stress pathway, activated when unfolded or misfolded proteins accumulate within the endoplasmic reticulum lumen. Inappropriately folded proteins can amass when large amounts of proteins are being synthesized and/or when cellular energy availability is not sufficient to correctly fold the proteins being trafficked into the endoplasmic reticulum (1, 2). A simple overview of UPR signaling is shown in Figure 1. The master regulator of UPR signaling, glucose-regulated protein 78 (*HSPA5*; GRP78), activates the UPR as a consequence of its release from the three UPR signaling controllers, PKR-like endoplasmic reticulum kinase (*EIF2AK3*; PERK), activating transcription factor 6 (*ATF6*), and inositol-requiring enzyme 1 (*ERN1*; IRE1). Under most normal conditions, PERK, ATF6, and IRE1 are held inactive in the endoplasmic reticulum membrane when bound to GRP78. Activated PERK phosphorylates eIF2α, resulting in the inhibition of cap-dependent protein translation and the induction of activating transcription factor 4 (*ATF4*) transcription. Increased ATF4 levels stimulate the activity of the pro-apoptotic DNA-damage inducible transcription factor 3 (*DDIT3*; also known as CHOP). When released from GRP78, ATF6 translocates to the Golgi complex where it is cleaved by site1/site2 proteases to form a highly active transcription factor. ATF6 induces the transcription of various other protein chaperones, including GRP78. Release of IRE1 from GRP78 enables IRE1 to oligermize and autophosphorylate. This activation of IRE1 enables its unconventional cytoplasmic splicing of X-box binding protein 1 (*XBP1*); the endonuclease activity of IRE1 removes a 26-base pair intron in XBP1 to produce its transcriptionally active form (XBP1-spliced, XBP1-S; Figure 2). XBP1-S increases the transcription of other protein chaperones, endoplasmic reticulum-associated protein degradation (ERAD), and inflammatory genes such as IL-6. Initial activation of UPR signaling is usually pro-survival, resulting in a reduced rate of protein translation and increased protein chaperone expression. These actions lessen the unfolded protein load within the endoplasmic reticulum and promote a restoration of cellular homeostasis. Prolonged activation of the UPR can promote cell death through CHOP signaling, when this or other prodeath signals dominate any remaining prosurvival activities (2).

## UPR Signaling in Breast Cancer

Breast cancer is the most prevalent cancer in women, with over 232,000 new cases of invasive breast cancer diagnosed annually within the United States of America. Furthermore, over 40,000 American women die from breast cancer every year (3). The most common breast cancer subtype, which comprises over 70% of all breast cancers, expresses the estrogen receptor-α (*ESR1*; ERα). These cancers are often treated with ERα targeted therapies, such as receptor antagonists (antiestrogens) including tamoxifen (TAM) or fulvestrant (ICI), or aromatase inhibitors like anastrozole or letrozole that inhibit ligand (17β-estradiol) production. About half of all ERα positive tumors respond to one of these first-line endocrine therapies, while the remainder is *de novo* resistant (4). Unfortunately, many initially responsive tumors develop resistance to these endocrine therapies and, overall, more women die from ER+ breast cancer than from any other molecular subtype (5). A better understanding of the molecular mechanism(s) of endocrine therapy resistance is urgently needed to design new and more effective interventions to prevent or reverse the emergence of drug resistance.

Possible molecular mechanisms of resistance to endocrine therapies include activation of the UPR pathway (2, 6, 7). Cancer cells have elevated UPR signaling that seems to promote survival in the glucose deprived and hypoxic tumor microenvironment, without a potent activation of the pro-apoptotic signaling resulting from prolonged UPR activation (8–10). GRP78 was shown to be elevated in all breast cancer subtypes [ERα, human epidermal growth factor receptor-2 (*ERBB1*; HER2) amplified, and triple negative] when compared with the normal surrounding breast tissue, indicating a critical role for GRP78 in driving breast tumorigenesis (11). GRP78 overexpression in both breast cancer cells and in breast tumors can confer anthracycline resistance (12). In addition, a preclinical report suggests a role for GRP78 in promoting estrogen-dependent breast cancer cell survival under estrogen-deprived conditions, simulating aromatase inhibitor activity. In this model, GRP78 inhibited the BCL2 family member, Bik (13). Through its binding of Bik, GRP78 prevents the activation of Bax/Bak thereby inhibiting apoptosis (14). We have also shown that knockdown of GRP78, using RNAi in ER+ breast cancer cells, reduces overall cellular levels of anti-apoptotic BCL2, BCL-W, and BCL-xL (11). GRP78 may also bind to pro-caspase-7, thereby preventing caspase-dependent apoptosis (15, 16). These data clearly illustrate a pro-survival role for GPR78 in breast cancer. Knockdown of GRP78 restored endocrine therapy sensitivity, while overexpression of GRP78 prevented tamoxifen effectiveness, further implicating GRP78 as a major regulator of endocrine responsiveness (2, 11).

XBP1 expression is widely reported in all breast cancer subtypes and correlates with poor prognosis (9, 10, 17). Expression of XBP1 with that of ERα suggests that XBP1 plays an important role in driving this subtype of breast cancer (18–20). In fact, Perou et al. included XBP1 expression among the key molecular components that may identify ER+ (luminal) breast cancers (21). We have shown that endocrine resistant breast cancer cell lines overexpress XBP1 (6), and that its overexpression confers this phenotype (22). In human breast cancer patients, XBP1 mRNA levels correlate with tamoxifen responsiveness, further supporting the role of XBP1 in endocrine therapy resistance (9). Targeting XBP1 through RNAi restored endocrine therapy sensitivity in resistant breast cancer cell lines (23). Overexpression of XBP1-S in MCF7 xenograft breast tumors rendered the tumors resistant to tamoxifen therapy. Moreover, Hu et al. showed that both isoforms of XBP1 (XBP1-U and XBP1-S) regulate NFκB activity by ERα-dependent mechanism, thereby linking XBP1/NFκB/ERα signaling. Furthermore, Hu et al. showed XBP1-S directly affects p65/RelA subunit of NFκB independent from ERα, indicating two different mechanisms by which XBP1 modulate NFκB signaling and cellular proliferation (23). Taken together, these data support the role of UPR signaling components modulating endocrine therapy responsiveness. In addition to being expressed in triple negative breast cancers (TNBC; ER-, PR-, HER2-) (10), XBP1 drives some TNBCs through modulating the HIF1α signaling pathway (24). For example, chromatin immunoprecipitation followed by ultra high throughput DNA sequencing (ChIP-seq) of XBP1 in MDA-MB-231 (a TNBC cell line) indicates a significant enrichment of HIF1α, suggesting co-localization of HIF1α and XBP1. Inhibition of XBP1 through transfection with shRNA in CD44^high^CD24^low^ breast tumor cells prevented paclitaxel and doxorubicin-mediated mammosphere formation. These data suggest that XBP1 mediates hypoxia-induced tumor cell survival in some TNBCs (24).

## Integration of UPR and Autophagy

**Figure 3:**
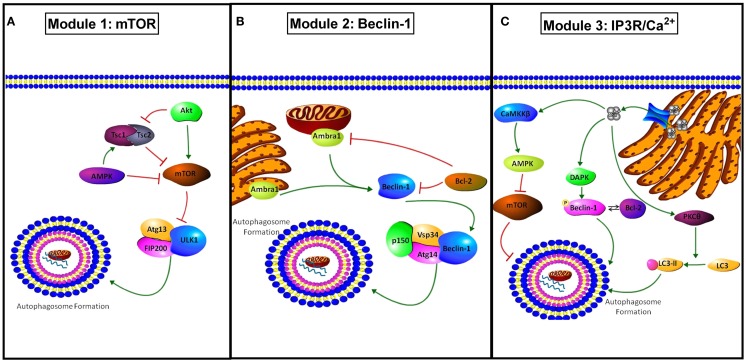
**Modules of autophagy initiation**. **(A)** Module 1 shows how mTOR inhibits autophagy through modulation of Atg13 and ULK1 inactivating the pre-autophagosomal structure (PAS)-initiation complex. AMPK and TSC1/2 inhibit mTOR activity, promoting autophagy, while active protein kinase B (AKT) promotes mTOR activation and concurrently inhibits TSC1/2 complex inhibiting autophagy. **(B)** Module 2 shows the regulation involved in BECN1 mediated autophagy. AMBRA1 present at both the mitochondria and endoplasmic reticulum promotes BECN1 activation and autophagy. BCL2 binds to BECN1 inhibiting activation of autophagy through the PI3K class 3 activation complex (PIK3C3, Vsp34, Atg14, p150, and beclin-1). BCL2 can also inhibit AMBRA1 when co-localized to the mitochondria. **(C)** Module 3 shows IP3R/Ca^2+^ regulation of autophagy. Ca^2+^ released from the EnR through the IP3R stimulates autophagy through activation of death associated protein kinase (DAPK) and the resulting phosphorylation of BECN1 that promotes its disassociation from BCL2. Regulation of autophagy by Ca^2+^ also occurs through stimulation of protein kinase C θ (PKCθ) promoting LC3 to LC3-II conversion, and through calcium/calmodulin-dependent protein kinase II beta (CAMK2B) activation resulting in AMPK stimulation and mTOR inhibition.

Autophagy is a cellular pathway of “self-eating” (25). Our group (among others) has implicated autophagy as a mechanism of therapeutic resistance in breast cancer (26–29). Autophagy involves the segregation of subcellular material into double membrane structures (autophagosomes) that then fuse with lysosomes (autolysosomes), wherein the cellular cargo is subsequently degraded by lysosomal hydrolases (30). This process maintains cellular integrity by the removal of aged, damaged, or unneeded organelles including mitochondria, Golgi complex, and endoplasmic reticulum. The autophagic pathway also supplements cellular metabolism by recycling the products of organelle degradation back into cell metabolism (2). Initiation of autophagic signaling can occur through three distinct molecular modules (Figure 3): (i) AMPK/mTOR, (ii) Beclin-1 (*BECN1*)/BCL2, and (iii) IP3R/Ca^2+^. In Module-1, activated mammalian target of rapamycin (*MTOR*) can inhibit Unc-51 like autophagy activating kinase 1 (*ULK1*). ULK1 forms a complex with autophagy related gene 13 (*ATG13*) and RB1-inducible coil-coil 1 (*RB1CC1*; also known as FIP200) that initiates autophagosome formation (31). In Module 2, autophagy/beclin-1 regulator 1 (AMBRA1) or BCL2 interacts with BECN1 to either promote (AMBRA1) or inhibit (BCL2) autophagosome formation (32). In the third module of autophagosome initiation, calcium release from the endoplasmic reticulum stimulates calcium/calmodulin-dependent protein kinase kinase 2 (*CaMKK2*), promoting the ability of AMP-activated protein kinase (*PRKAA1*; AMPK) to inhibit the autophagic repression regulated by mTOR signaling. Concurrently, calcium release activates death associated protein kinase (*DAPK*) to activate BECN1. Also, calcium stimulates protein kinase C-θ (*PRKCQ*; PKCθ) to promote the lipidation of microtubule associated protein 1 light chain 3 (*MAP1LC3*; LC3-II) promoting autophagic activation (33). Therefore, cellular environmental changes such as nutrient deprivation, hypoxia, or therapeutic intervention may stimulate autophagosome formation.

While autophagy is often downregulated early in initial tumorigenesis, autophagy is frequently upregulated in its later stages (2). This upregulation of autophagy has been shown to combat the harsh negative environmental conditions in which most solid tumors exist. However, conflicting reports define autophagy as a “double-edge sword,” where autophagic activation may be either pro-apoptotic or pro-survival (34, 35). Bursch et al. dosed MCF-7 (estrogen dependent, ER+ breast cancer cell line) with various antiestrogen therapies and showed increased autophagosome formation, and theorized that endocrine therapies promote autophagy-mediated cell death (36). However, Samaddar et al. suggested that what Bursch et al. observed was a failed attempt by the cell to use autophagy to survive due to increased autophagy observed in the surviving TAM treated MCF-7 cell population (27). These authors then hypothesized that the duality of autophagic signaling (either promotes survival or cell death) may be dependent on the quantity of cellular autophagosomes, suggesting a threshold limit. Samaddar et al. also showed that inhibition of autophagy through Beclin1 siRNA transfection or using a chemical inhibitor of autophagy, 3-methyladenine (3-MA), significantly potentiated antiestrogen-induced cell death (27). We have shown elevated levels of lipidated autophagic protein LC3-II in antiestrogen resistant breast cancer cells when compared with their therapy sensitive parental cell line, suggesting increased basal autophagosome formation in endocrine resistant breast cancer cells (11). Furthermore, inhibition of autophagy through ATG5 or ATG7 silencing potentiates antiestrogen responsiveness in ER+ breast cancer cell lines (37). Mice bearing antiestrogen-resistant LCC9 and MCF-7RR orthotopic xenografts were treated with a chemical inhibitor of autophagy, chloroquine (CQ), and the results showed that inhibition of autophagy *in vivo* restored tamoxifen sensitivity to resistant tumors (38). Inhibition of mitochondria specific autophagy (mitophagy) through PTEN inducible putative kinase 1 (*PINK1*) silencing restored antiestrogen sensitivity to resistant breast cancer cell lines (39). Moreover, endocrine-resistant breast cancer cell lines express elevated parkin RBR E3 ubiquitin protein ligase (*PARK2*; parkin) protein levels, a protein critical for the induction of mitophagy (40). These data suggest that mitochondrial specific autophagy also plays a key role in the development and maintenance of antiestrogen therapy resistance in ER+ breast cancer.

Unfolded protein response and autophagy are linked through several mechanisms. For example, interactions between the PERK/e1F2α axis of UPR result in the cleavage of LC3 and stimulation of autophagy. Also, ATF4 promotes autophagy through transcription of ATG12. Another arm of UPR, involving the kinase IRE1, activates autophagy via mitogen-activated protein kinase (MAPK) c-Jun terminal kinase (JNK) stimulation (1, 30, 41). While multiple UPR pathways regulate autophagy, Ogata et al. demonstrated, through siRNA experiments in a neuroblastoma cell line, that endoplasmic reticulum stress induced autophagy is the result of IRE1-JNK activation, not ATF6 or PERK (42). However, Li et al. showed that silencing of GRP78 in embryonic kidney cells and ovarian cancer cells prevented the stimulation of autophagy and the formation of autophagosomes (43). In addition, this group showed that XBP1 knockdown in these cells had no effect on basal levels or the stimulation of autophagosome formation, suggesting a critical link between GRP78, autophagy, and survival independent of UPR (43).

We have established a novel link between GRP78 and AMPK to regulate autophagy. We showed that overexpression of GRP78 in the endocrine sensitive cell line LCC1 resulted in the formation of autophagosomes and promoted autophagic flux (11). Stimulation of autophagy mediated by GRP78 was inhibited by tuberous sclerosis-2 (*TSC2*) or AMPK silencing, demonstrating that GRP78 modulates AMPK signaling to effect autophagosome formation (11). Moreover, silencing of GRP78 in breast cancer cell lines decreased pro-survival BCL2 family members with no effects on BECN1 levels, suggesting that GRP78 does not directly modulate BECN1 to affect autophagy (11). However, BCL2 can sequester BECN1 and GRP78-mediated changes in BCL2 expression could indirectly regulate autophagy (2). Importantly, GRP78, IRE1, and PERK can independently stimulate autophagy in a cell context specific manner. The integration of UPR and autophagy is intuitively rational; the accumulation of unusable unfolded proteins should trigger a means to concurrently clear or fix these proteins, and/or recycle their components to aid in resolving the stress that originally caused these proteins to accumulate.

## Estrogen Signaling in Antiestrogen Resistance

Over 70% of all breast cancers express ERα, and therefore, respond to estrogens. The growth and proliferation of ERα positive breast tumors are predominately driven by the receptor’s ligand, estrogen. Drugs targeting either the receptor (tamoxifen or fulvestrant) or the ligand (aromatase inhibitors) have been successful at treating this disease. Unfortunately, many of these tumors acquired resistance and recur as endocrine insensitive breast cancers. Therefore, understanding the ER signaling network and the permutations that derive from the antiestrogen resistance phenotype is critical to make progress in the treatment of advanced breast cancers. Many growth factor signaling pathways are implicated in the development of resistance including MAPK, phosphoinositol 3-kinase (PI3K), epidermal growth factor (EGF), and insulin-like growth factor (IGF) 1 receptor signaling (44). Also, various mutations in ERα were theorized to confer endocrine therapy independence to cells, as recently reviewed in Ref. (45). Various clinical studies have indicated only probably less than 25% of metastatic breast tumors harbor ERα mutations that may drive resistance (46, 47), suggesting perturbations in growth factor or other molecular signaling pathways as major component mediating endocrine independence. Mathematical modeling offers a dynamic “road-map” perspective of integrating the behavior of cells with often contradicting signaling components to create a molecular interaction graph (48). These models can design the transitions between endocrine therapy sensitive and resistant states in breast cancer (49), the switch between estrogen receptor and growth factor signaling (50), and model in the interactions between ERα and UPR signaling (37), highlighting the usefulness of integrating mathematical modeling into cancer research.

## Estrogen, the Estrogen Receptor, and Regulation of UPR and Autophagy

While estrogens primarily acting through ERα stimulate breast cancer cell survival and growth, ERα-targeted therapies such as tamoxifen or fulvestrant (ICI) stimulate pro-survival UPR and autophagy signaling (37). We previously showed that the selective estrogen receptor downregulator (SERD) ICI stimulates UPR signaling in both antiestrogen sensitive and resistant breast cancer cell lines. However, we found a timing differential in the stimulation of UPR signaling between endocrine therapy sensitive and resistant cell lines. UPR was activated in the resistant LCC9 cells, 48–96 h before the LCC1 antiestrogen sensitive parental cell line, suggesting a possible link between the timing of pathway stimulation and endocrine resistance (37). Silencing ERα through RNAi inhibited UPR signaling components while concurrently stimulating autophagy (37). These data indicate that endocrine-targeted therapies may act through the ability of ERα to control autophagy but not directly to control UPR signaling. Moreover, in accordance to other reports, knockout of ERα resulted in the re-sensitization of resistant breast cancer cell lines to endocrine-targeted therapies (51). A report describing the effects of bortezomib (a proteasome inhibitor) in MCF7 breast cancer cells demonstrated that ICI increased the aggregation of ERα in the cytoplasm and increased UPR signaling, increasing proteasome inhibitor-mediated cell death (52). We showed that ICI increased cytoplasmic aggregation of ERα, and that endocrine resistant LCC9 cells have elevated basal levels of cytoplasmic ERα, when compared with their therapy sensitive parental cells, likely explaining the increased UPR signaling observed in these cell lines (53). Knocking out ERα prevented both antiestrogen-mediated cytoplasmic aggregation of ERα and UPR activation. Moreover, we showed that inhibiting UPR signaling by ERα silencing prevented NRF2-mediated cellular antioxidant response and increased the concentration of reactive oxygen species (ROS) that led to more cell death (37). These data indicate that ERα can regulate UPR and autophagy through various distinct mechanisms: ERα activity, ERα localization, and through increased ROS production.

Estrogen induces increased protein translation that may also stimulate UPR signaling. We showed that higher concentrations of 17β-estradiol induce CHOP signaling in both LCC1 and LCC9 breast cancer cells, suggesting estrogen may induce UPR signaling (37). Recently, Andruska et al. demonstrated that 17β-estradiol, acting through an extranuclear ERα-mediated event, stimulates PIP_2_ to DAG conversion releasing cytoplasmic 1,4,5-triphosphate (IP_3_) through PLC-γ activation. IP_3_ stimulates the release of calcium from the endoplasmic reticulum and promotes UPR signaling and cellular proliferation (54). These data describe a novel role of estrogen and ERα in stimulation of UPR signaling.

The other estrogen receptor, estrogen receptor-β (*ESR2*; ER-β), was recently shown to effect UPR signaling. ER-β inhibits the growth of breast cancer cells directly opposing the proliferative actions of ERα (55). Furthermore, ER-β reduced breast cancer cell invasiveness, suggesting a role of ER-β in metastasis (55, 56). Clinical breast cancer data sets reported an association between better prognosis and ER-β expression (57). Recent work by Rajapaksa et al. indicates a novel reciprocal relationship between ER-β and XBP1 (58). ER-β stimulates synoviolin1 (SYVN1) resulting in the degradation of IRE1, thereby reducing XBP1 splicing and activity (58). Reduction of XBP1 by ER-β is a novel mechanism by which ER-β inhibits breast cancer cell growth.

## UPR and Cellular Energy Sensing Mechanisms

GRP78, or glucose-regulated protein 78, is aptly named due to its regulation by cellular glucose and energy levels. Along with GRP78, there are other glucose-regulated proteins in the family, such as GRP94 (*HSPA90B1*), GRP170 (*HYOU1*), GRP75 (*HSPA9*; mortalin), and GRP58 (*PDIA3*) (59). As with GRP78, most of these other glucose regulated proteins are found elevated in antiestrogen resistant breast cancer cells (Table 1), giving further evidence of the importance of these proteins in modulating therapeutic responsiveness. While GRP78, GRP170, and GRP94 are endoplasmic reticulum protein chaperones that play a role in UPR signaling, the other GRPs are either located in other cellular organelles or their molecular actions dramatically differ. For example, GRP75 is predominately located in the mitochondria and plays a role in longevity and mitochondrial protein transport (60), GRP58 is located in the endoplasmic reticulum lumen and is a protein disulfide isomerase that is critical to the peptide loading process of major histocompatibility complex (MHC) class I pathway (61). Further investigation into these other GRP’s is currently ongoing to determine their role in endocrine therapy resistance in ER+ breast cancers.

**Table 1:** **Glucose-regulated proteins in antiestrogen resistance**.

Glucose regulated proteins	Gene name	Gene fold change LCC9 vs LCC1	Gene fold change MCF7RR vs MCF7
GRP94	HSP90B1	1.24	Non-significant
GRP78	HSPA5	1.89	1.51
GRP75	HSPA9	1.52	2.23
GRP170	HYOU1	Non-significant	1.57
GRP58	PDIA3	1.43	1.32

*Fold change mRNA levels of GRP78, GRP94, GRP170, GRP58, and GRP75 in antiestrogen sensitive vs resistant ER+ breast cancer cell lines*.

We previously showed the novel regulation of GRP78 on regulating AMPK and mTOR signaling to control autophagy in breast cancer cells (11, 62). Others have linked GRP78 and AMPK signaling in leukemia and ovarian cancer cells (63, 64). AMPK activating compounds, such as metformin and 5-Aminoimidazole-4-carboxamide ribonucleotide (AICAR), were shown to increase GRP78 protein levels in acute lymphoblastic leukemia cell lines, further supporting the molecular interaction between AMPK and GRP78 signaling (64, 65). Vaspin, an adipokine, was shown to bind to cell surface GRP78, creating a vaspin-GRP78-MTJ1 complex that was shown to activate AMPK in H-4-II-E-C3 cells. Pretreatment with a GRP78 antibody blocked the formation of the vaspin complex and prevented AMPK activation, indicating a critical role of cell surface GRP78 in AMPK activation (66). AMPK is a critical nutrient and energy sensor activated when cellular ATP levels are insufficient. AMPK is also a driver of systemic energy balance through modulating metabolism, circadian rhythm, and feeding behaviors (67). Therefore, it is not surprising that UPR signaling and endoplasmic reticulum stress may impact the development of various metabolic diseases such as diabetes, obesity, and insulin resistance (68–71). UPR signaling is elevated in the adipocytes of obese mice and obese non-diabetic humans (70, 72). Previously, obese subjects that lost weight following gastric bypass surgery exhibited significantly lower endoplasmic reticulum stress signaling components, when compared with obese individuals, in their subcutaneous fat and liver tissues (73). GRP78 heterozygous mice are resistant to diet-induced obesity (74). XBP1 deficiency prevented obesity in mice fed a high fat diet but XBP1 deficient mice developed insulin resistance (68). PERK homozygous deletion in mice is perinatal lethal due to the development of diabetes consistent with β-cell defects (75, 76). These studies indicate the possible roles of UPR signaling in metabolic signaling pathways and diseases.

## Therapeutic Agents Modulating UPR Signaling

ERα targeting therapies, such as tamoxifen and fulvestrant, stimulate UPR signaling resulting in breast cancer cell survival (37). Addition of the proteasomal inhibitor, bortezomib, with fulvestrant in ER+ breast cancer cells resulted in markedly increased UPR signaling promoting apoptosis (52), suggesting a threshold limit wherein the duration of UPR signaling determines cell survival or death. ER-β agonists may be used to modify UPR signaling. ER-β activation was shown to inhibit IRE1, thereby reducing XBP1 splicing in breast cancer cell lines (58). Modified bacterial toxins, prunustatin A(1) and versipelostatin, were shown to cleave and inactivate GRP78 (77, 78). Humanized antibodies against GRP78 (PAT-SM6) have been developed and are currently in clinical trials to determine whether targeting cell surface GRP78 may be an effective anti-cancer treatment (16). Moreover, natural products such as the soy phytoestrogen genistein, curcumin, and the green tea polyphenol EGCG have been shown to inhibit GRP78 (16), indicating a possible role of dietary interventions modulating UPR signaling. Therapeutic agents targeting UPR in preclinical models indicate a great potential for the treatment of advanced breast cancer. Combining current breast cancer treatment modalities with drugs that target UPR signaling may decrease tumor formation, tumor size, distant metastases, and/or the development of drug resistance. Further experimentation is critical to determine the effectiveness of UPR targeting drugs and successful combinatorial drugs regimens; however, preclinical data are promising.

## UPR and the Tumor Microenvironment

The predominant focus of many studies is on the molecular signaling in tumor epithelial cells. However, breast tumors contain many different cell types including endothelial cells, fibroblastic cells, macrophages, and T-cells that integrate to form a complex bio-infrastructure that generally supports neoplastic epithelial cell growth (79). Understanding how the UPR may affect these other cell types is critical to the design of therapeutics and the treatment of breast cancer. A study by Dong et al. showed that breast tumors generated in GRP78 heterozygous mice were smaller than wild-type tumors and displayed decreased tumor microvasculature (80, 81). Further studies indicated that GRP78 silencing in endothelial cells reduced endothelial tube formation and inhibited endothelial cell migration, suggesting that GRP78 plays a critical role in angiogenesis (80, 81). Previous studies by Romero-Ramirez et al., and later confirmed by Chen et al., showed the link between XBP1 and hypoxia (24, 82). A major difference between these studies is their conclusions on the effects of XBP1 on angiogenesis. Inhibition of XBP1 in an embryonic fibroblast cell line and in fibrosarcoma cells had no effect on the secretion of pro-angiogeneic growth factors, such as vascular endothelial growth factor (VEGF) and basic fibroblast growth factor (bFGF). Thus, Romero-Ramirez et al. concluded that the molecular mechanism controlling tumor growth deficiency in XBP1-inhibited cells is likely hypoxia sensitivity and not an inhibition angiogenesis (82). However, in a later publication, these authors showed that XBP1 inhibition reduced angiogenesis in a pancreatic adenocarcinoma model, suggesting that the effects of XBP1 on angiogenesis may be cell type dependent (83). In Chen et al., inhibition of XBP1 in MDA-MB-231 breast cancer cells also resulted in decreased tumor blood vessel density as determined by CD31 immunoreactivity (24). These data strongly suggest a cell context specific role for UPR signaling in mediating tumor angiogenesis.

Increased extracellular matrix (ECM) collagen deposition was associated with breast tumor progression and chemotherapy resistance (79, 84). Studies in a preclinical setting demonstrate that targeting cancer-associated fibroblasts and collagen are an effective therapeutic option in the treatment of breast tumors (85, 86). Epithelial to mesenchymal transition (EMT) also correlates with breast tumor progression and endocrine therapy resistance (84). EMT is associated with an increase in cellular protein secretion; therefore, the recent study highlighting the link between EMT and UPR reached a logical conclusion (87). Feng et al. demonstrated that EMT favorably stimulated the PERK/ATF4 signaling axis of UPR (87). This EMT-mediated stimulation of PERK was required for cancer cells to invade and metastasize (87). Recent studies have implied a possible role of GRP78 in EMT (16, 88, 89). Knockdown of GRP78 through RNAi led to decreased cell mobility, invasion, and ECM degradation in hepatocellular carcinoma cells (88). Furthermore, GRP78-mediated colon cancer metastasizes through regulation of EMT markers by ROS/NRF2 signaling (89). XBP1 was also shown to regulate EMT through modulation of snail family zinc finger 1 (*SNAI1*; snail). Snail binds to and inhibits e-cadherin, thereby promoting EMT. Inhibition of XBP1 inhibited snail, while concurrently increasing e-cadherin expression (90). Overexpression of XBP1 increased snail protein, reduced e-cadherin levels, and increased mesenchymal markers in breast cancer cells (90). These data highlight an important role of UPR signaling in the tumor microenvironment and suggest that blocking the UPR in cancer could treat both the tumor epithelial cells and also pro-survival activities within the tumor microenvironment.

## Conclusion and Future Directions

The unfolded protein response signaling integrates many different cellular processes, including protein folding, rates of transcription and translation, protein degradation, autophagy, metabolism, and cell fate pathways. Breast cancer tumors have elevated UPR signaling components, including GRP78 and XBP1, which drive endocrine therapy resistance. Treatment of breast cancer cell lines and tumors with ERα targeting therapies results in the activation of pro-survival UPR signaling, suggesting an intrinsic resistance mechanism. Designing drugs, which may target pro-survival UPR components (e.g., IRE1 or GRP78), while activating pro-apoptotic UPR signaling (CHOP) would be highly beneficial for the treatment of these cancers. Estrogen and ERα both serve as a central regulator of autophagy and UPR modulating these signaling pathways through different molecular mechanisms. Antiestrogen therapies result in the accumulation of dysfunctional ERα in the cytosol, leading to UPR activation, while endocrine therapies direct inhibition of ERα activity promotes autophagosome formation. Both the stimulation of autophagy and UPR by antiestrogens promote survival and resistance. Recently, some studies are investigating the role of UPR signaling in the tumor microenvironment. Of particular interest is the critical role of UPR signaling in immunity, highlighting the importance of syngeneic models in developing UPR-targeting therapeutic strategies. While targeting UPR may be beneficial to inhibit tumor epithelial cells, the effect on innate and adaptive immunity is less clear. Further studies are needed to clarify the role of UPR in T-cell signaling and macrophage cytolytic capacity. Other studies showed UPR signaling promotes changes within the tumor microenvironment that favors migration, metastasis, and invasiveness, clearly demonstrating why targeting UPR would be an effective therapeutic option for the treatment of breast cancer.

## Author Contributions

RC and KC both wrote and edited the manuscript.

## Conflict of Interest Statement

The authors declare that the research was conducted in the absence of any commercial or financial relationships that could be construed as a potential conflict of interest.
